# Rewilding in the face of climate change

**DOI:** 10.1111/cobi.13531

**Published:** 2020-06-01

**Authors:** Carlos Carroll, Reed F. Noss

**Affiliations:** ^1^ Klamath Center for Conservation Research Orleans CA 95556 U.S.A.; ^2^ Florida Institute for Conservation Science Melrose FL 32666 U.S.A.

**Keywords:** climate adaptation, climate velocity, connectivity, conservation planning, refugia, protected areas, adaptación climática, áreas protegidas, conectividad, planeación de la conservación, refugios, velocidad climática, 气候适应, 气候变化速率, 连接度, 保护规划, 避难所, 保护区

## Abstract

Expansion of the global protected‐area network has been proposed as a strategy to address threats from accelerating climate change and species extinction. A key step in increasing the effectiveness of such expansion is understanding how novel threats to biodiversity from climate change alter concepts such as rewilding, which have underpinned many proposals for large interconnected reserves. We reviewed potential challenges that climate change poses to rewilding and found that the conservation value of large protected areas persists under climate change. Nevertheless, more attention should be given to protection of microrefugia, macrorefugia, complete environmental gradients, and areas that connect current and future suitable climates and to maintaining ecosystem processes and stabilizing feedbacks via conservation strategies that are resilient to uncertainty regarding climate trends. Because a major element of the threat from climate change stems from its novel geographic patterns, we examined, as an example, the implications for climate‐adaptation planning of latitudinal, longitudinal (continental to maritime), and elevational gradients in climate‐change exposure across the Yellowstone‐to‐Yukon region, the locus of an iconic conservation proposal initially designed to conserve wide‐ranging carnivore species. In addition to a continued emphasis on conserving intact landscapes, restoration of degraded low‐elevation areas within the region is needed to capture sites important for landscape‐level climate resilience. Extreme climate exposure projected for boreal North America suggests the need for ambitious goals for expansion of the protected‐area network there to include refugia created by topography and ecological features, such as peatlands, whose conservation can also reduce emissions from carbon stored in soil. Qualitative understanding of underlying reserve design rules and the geography of climate‐change exposure can strengthen the outcomes of inclusive regional planning processes that identify specific sites for protection.

## Abstract 2

*Article Impact Statement*: Rewilding concepts updated to consider climate change increase mitigation and biodiversity conservation gain from protected area expansion.

## Abstract (es)

Retorno a la Vida Silvestre de frente al Cambio Climático

**Resumen**

La expansión de la red mundial de áreas protegidas ha sido propuesta como una estrategia para tratar con las amenazas del creciente cambio climático y la extinción de especies. Un paso importante para el incremento de la efectividad de dicha expansión es el entendimiento de cómo las amenazas novedosas para la biodiversidad que provienen del cambio climático alteran conceptos como el retorno a la vida silvestre, el cual ha apuntalado muchas propuestas de grandes reservas interconectadas. Revisamos los obstáculos potenciales que representan el cambio climático para el retorno a la vida silvestre y encontramos que el valor de conservación de las áreas protegidas grandes persiste bajo el cambio climático. Sin embargo, se le debería brindar mayor atención a la protección de los microrefugios, del gradiente ambiental completo y de las áreas que conectan climas adecuados actuales y futuros. También se le debe brindar atención al mantenimiento de los procesos ambientales y a la estabilización de la retroalimentación por medio las estrategias de conservación que son resilientes a la incertidumbre relacionada con las tendencias climáticas. Ya que un elemento principal de la amenaza que representa el cambio climático surge de sus patrones geográficos novedosos examinamos, como ejemplo, las implicaciones de los gradientes latitudinales, longitudinales (continental a marítima) y altitudinales para la planeación de la adaptación climática dentro de la exposición al cambio climático en toda la región de Yellowstone a Yukón, el sitio de una propuesta icónica de conservación diseñada inicialmente para conservar especies carnívoras de amplia distribución. Además de un énfasis continuo sobre la conservación intacta del paisaje, se requiere la restauración de las áreas degradadas de baja elevación dentro de la región para capturar los sitios importantes para la resiliencia climática a nivel de paisaje. La exposición climática extrema proyectada para la parte boreal de América del Norte sugiere que se necesitan metas ambiciosas para la expansión de la red de áreas protegidas que se encuentran allí para incluir también a los refugios creados por la topografía y las características ecológicas, como las turberas, cuya conservación también puede reducir las emisiones de carbono almacenado en el suelo. El entendimiento cualitativo de las reglas subyacentes de diseño de reservas y la geografía de la exposición al cambio climático puede fortalecer los resultados de los procesos de planeación regional incluyente para identificar los sitios específicos que requieren protección.

## 摘要

目前已有提议将扩大全球保护区网络作为应对不断加剧的气候变化及物种灭绝威胁的一项战略。然而, 提高该措施有效性的关键在于了解气候变化对生物多样性的新威胁将如何改变一些重要概念, 比如支持着许多大型连通保护区建立的 “野生动物恢复” 概念。本文描述了气候变化对野生动物恢复的潜在挑战, 发现在气候变化的情况下, 大型保护区仍然具有保护价值。尽管如此, 还是应更多地注意保护微型避难所、大型避难所、完整的环境梯度以及连接当前和未来适宜气候区的区域。此外, 应通过对气候趋势不确定性具有恢复力的保护战略来维持生态系统过程和反馈。由于气候变化威胁的一个主要因素来自其产生的新地理模式, 因此我们以美国黄石到育空地区为例, 分析了气候变化背景下经度 (从陆地到海洋) 、维度和海拔梯度上气候适应规划的影响, 该地区具有为保护广泛分布的食肉动物而设计的标志性保护方案。我们发现, 除了继续强调保护完整景观外, 还需要恢复该地区退化的低海拔地区, 以建立具有重要的景观尺度气候恢复力的位点。对北美北部地区的极端气候预测表明, 有必要制定更具雄心的目标, 扩大该地区的保护区网络, 以纳入由地形和生态特征建立的避难所, 例如泥炭地, 其保护还可以减少储存在土壤中的碳的排放。定性理解潜在的保护区设计规则和气候变化暴露下的地理位置, 有助于加强确定具体保护位点的大范围区域规划过程。 **【翻译: 胡怡思; 审校: 聂永刚】**

## Introduction

The twin crises created by the accelerating pace of climate change and loss of biodiversity (IPBES 2019) have prompted calls for expansion of the terrestrial protected area network. For example, a recent proposal for a Global Deal for Nature suggests that 30% of the terrestrial landscape be formally protected by 2030 and an additional 20% designated as climate stabilization areas, which would maintain or increase the carbon stored in vegetation and soil (Dinerstein et al. 2019). Rather than focusing solely on percentage targets, conservation scientists have also called for more effective placement of new protected areas so that they contribute maximally to reducing biodiversity loss and mitigating climate change (Visconti et al. 2019).

Such proposals are not entirely new. The concept of rewilding, a key element underpinning the call for expanded protected area networks, was defined by Soulé and Noss (1998) as restoration and protection of interconnected wilderness landscapes large enough to support wide‐ranging mammals, goals the proponents termed the 3Cs (cores, corridors, and carnivores). Reserve network design in the context of rewilding overlaps with conservation planning in the general sense but emphasizes ambitious goals for protection of large connected core areas that suit the needs of focal species with large area requirements. In contrast to most of conventional conservation planning, rewilding attempts to recover the fundamental properties of wilderness landscapes, including complete food webs and natural disturbance regimes (Soulé & Noss 1998).

Although elements of the concept can be found in earlier writings by Victor Shelford and others (Croker 1991), rewilding framed this wilderness recovery goal within the context of modern conservation science. The rewilding framework is increasingly relevant given the ambitious targets for expanded protected area networks proposed by scientists, civil organizations, intergovernmental bodies, and some national governments. For example, the Canadian government has endorsed the Convention on Biological Diversity goal of 30% protection by 2030 (CBD 2020), and Bhutan has placed over 42% of its land in protected areas (Locke 2014).

While rewilding initially focused on questions concerning landscape structure and reserve design, subsequent development of the concept, especially in Europe, shifted focus toward techniques for restoration of ecosystem processes in formerly human‐modified landscapes (e.g., by reestablishing populations of large, usually semidomesticated, herbivores) (Lorimer et al. 2015; Perino et al. 2019, Pettorelli 2019). Ecosystems have structural, functional, and compositional components, all of which interact to determine biodiversity (Noss 1990). Both the structural and process‐oriented definitions of rewilding are necessary and complementary because exclusive emphasis on process could result in unacceptable losses of species sensitive to anthropogenic change. We use the term “rewilding” in the original structure‐oriented definition throughout the remainder of the article.

Although ecologists had begun to consider the implications of climate change for reserve design by the mid‐1980s (Peters & Darling 1985), the topic received only passing mention in the initial writings on rewilding (Soulé & Noss 1998; Soulé & Terborgh 1999). The concept's proponents realized that climate change provided additional support for ambitious conservation goals, but they did not have sufficient information to assess how this increasing threat would influence the design of protected area networks. Although previous reviews have considered climate change within the process‐oriented definition of rewilding (Seddon et al. 2014; Corlett 2016; Perino et al. 2019) or in the general context of protected area management (Jones et al. 2016; Reside et al. 2018), we focused on the less‐treated question of how climate change can be incorporated within rewilding's structure‐oriented focus on design of large interconnected reserve networks.

**Figure 1:**
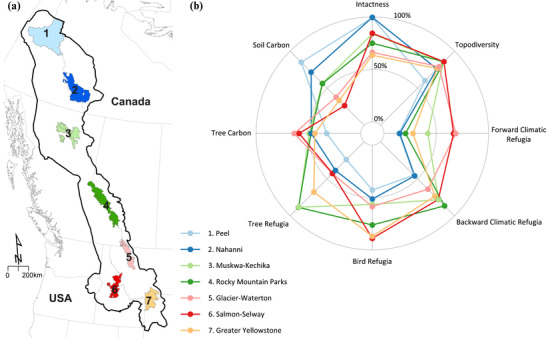
(a) Yellowstone‐to‐Yukon region showing the 7 largest protected areas (or protected‐area planning region for Peel watershed, 1) and (b) comparison of values of the 8 climate adaptation metrics for these protected areas. All data are scaled to equal‐area quantiles for comparability. Data sources are in Supporting Information.

We first explored whether and how the threat to biodiversity from climate change prompts reconsideration of the central concepts of rewilding, the 3Cs framework, and other ambitious strategies for expansion of protected areas. Such a review can serve as the initial element of a three‐stage process in which practitioners first evaluate basic reserve design rules and then qualitatively consider the geography of climate change exposure (e.g., spatial patterns created by the interaction of global climate systems with regional topography). This qualitative understanding of threat patterns can inform a final stage in which systematic conservation planning methods are used to identify specific sites for protection (Stralberg et al. 2020*b*). We considered how practitioners can incorporate such a climate‐informed rewilding strategy in regional conservation planning processes with recently developed spatial data projecting future exposure and vulnerability of biodiversity to climate change in the Yellowstone‐to‐Yukon (Y2Y) region (Fig. 1a) (Carroll et al. 2017; Stralberg et al. 2020b, 2018).

## Cores, Corridors, and Carnivores in the Context of Climate Change

### Effects of core reserve size on carnivores and other focal species

Design of protected area networks has historically been informed by principles inspired by the theory of island biogeography (Wilson & MacArthur 1967). Diamond (1975) summarized these principles as implying that the effectiveness of protected areas in conserving biodiversity increases as they became bigger, more connected, and more circular (i.e., with less edge). Large carnivores are among the species with the largest area requirements to sustain viable populations (Shaffer 1981). Soulé and Noss (1998) proposed that protected area networks large enough to sustain focal carnivore species will also be of sufficient size to conserve species with smaller area requirements (Table 1); this is the familiar umbrella species strategy (Roberge & Angelstam 2004).

**Table 1:** Four central concepts underlying reserve design and potential challenges to application of these concepts due to climate change

Concept	Rationale in conservation science, including rewilding and 3Cs^*^	Factors potentially reducing relevance under climate change	Factors potentially maintaining or increasing relevance under climate change
Reserve size—bigger is better	Larger reserves show increased ability to support populations of carnivore umbrella species.	Representation of gradients is more important than reserve size. Assuming no increase in total protected area, small dispersed reserves are best.	Viable carnivore populations may help buffer effects of climate change. Multiscale networks that include both micro‐ and macrorefugia are necessary. This implies the need for enlargement of the protected area network.
Reserve shape—less edge is better	Edge effects alter ecosystem processes and may lead to loss of interior species.	Benefits of capturing climatic gradients outweigh the drawbacks of increased edge.	Edge effects (drought, fire) are accentuated under climate change, implying the need for an expanded protected area network that captures gradients within large core areas.
Connectivity—more connected is better	Habitat connectivity allows a network of protected areas to be large enough to sustain viable populations.	Assisted colonization is more relevant than conserving natural habitat connectivity for those species which will not be able to track suitable climate via dispersal.	External refugia (areas of accessible climatically suitable habitat) have a role for most species except the least vagile. Sea‐level rise accentuates need for connectivity between coastal and inland areas.
Ecological processes—conserve and restore	Conservation of species and ecosystems requires restoring ecological processes to wild or (in North America) pre‐European settlement status.	Historical baselines are no longer relevant to restoration under climate change.	Restoration of processes can increase ecosystem resilience to climatic stressors.

^*^The 3Cs refers to use of Cores, Corridors, and Carnivores as a conservation planning framework.

In addition to their role as umbrella species, intact assemblages of carnivores may help buffer the effects of climate change on other species, for example by maintaining availability of carrion despite a shift towards shorter seasonal duration of snow cover, which preserves carrion (Wilmers & Getz 2005). Trophic cascades determined by the abundance of large carnivores may also influence net ecosystem productivity and hence carbon cycling, although the direction and magnitude of this effect varies between ecosystems (Wilmers & Schmitz 2016). The 3Cs approach is not invalidated by projections that even the largest reserves may not retain carnivore species such as the wolverine (*Gulo gulo*), a species highly vulnerable to climate change via loss of snow‐covered denning habitat (McKelvey et al. 2011). Given adequate broad‐scale connectivity, large protected areas can sustain other area‐dependent focal species adapted to their future climates as well as species with broader climatic tolerances such as the gray wolf (*Canis lupus*) (Carroll et al. 2001). Although the 3Cs approach uses carnivore area requirements to inform reserve proposals, in more developed ecoregions that lack sufficiently large natural areas to sustain large carnivores, the focal species element of the 3Cs strategy can be informed by area requirements of smaller focal species that are also vulnerable to changes in habitat configuration (Noss & Cooperrider 1994).

More recent reviews have concluded that a group of small reserves may be more effective than a large single reserve under changing climates if the former encompasses broader climatic and elevational gradients (Table 1) (Pearson & Dawson 2005; Araújo 2009). Because the best option between a single large and several small reserves will largely depend on the specific planning context and species of concern, this argument, like the single large or several small debate as a whole, may have limited practical relevance. One aspect of the question, however, is uniquely relevant under climate change: the necessity of including both macro‐ and microrefugia within reserve networks.

Microrefugia (small areas with locally favorable environments within otherwise unsuitable climates) may be important to persistence of species with modest area requirements under climate change, especially in topographically complex landscapes (Dobrowski 2011). The North American protected area network is predominantly located in low productivity areas (Scott et al. 2001), which are often at high elevations, so existing protected areas may have higher microrefugia potential than expected by chance (Oldfather et al. 2020). However, many microrefugia form holdout or stepping‐stone habitat (Hannah et al. 2014), which has only transient value before being overwhelmed by broad‐scale climate shifts. Therefore, a robust conservation network should include both microrefugia and large reserves that capture macrorefugia (areas where broad‐scale climate is relatively stable and suitable for persistence) (Carroll et al. 2017) (Table 1). Area requirements for capturing macro‐ and microrefugia and broad environmental gradients may equal or exceed the area required to maintain focal species populations in the absence of climate change (Carroll et al. 2017; Stralberg et al. 2020*b*).

### Effects of reserve shape

The effect of increased edge on persistence of species and ecosystem processes has been documented in many systems (Ries et al. 2004), leading to the principle that compact (circular) reserves are more effective at retaining elements of biodiversity of conservation concern (e.g., species associated with interior forest or vulnerable to human exploitation) (Diamond 1975) (Table 1). Nevertheless, subsequent reviews suggested that under climate change, a more linear reserve that spans climatic and elevational gradients may be more effective than a compact reserve (Table 1) (Pearson & Dawson 2005; Araújo 2009). Proponents of rewilding also proposed protection of intact elevational and latitudinal gradients to facilitate upslope or poleward migration of species in response to climate change (Noss & Cooperrider 1994). The contrast between the two perspectives hinges on the fact that rewilding proponents viewed novel stressors such as climate change as altering the role, but confirming the value of large core reserves (including those spanning climatic gradients) as anchors of regional protected area networks.

This latter perspective is supported by evidence that edge effects in small or linear reserves may be accentuated by the projected increase in extreme events such as droughts or megafires under climate change and the increased sensitivity of areas of forest near edges to these changes (Noss 2001) (Table 1). In regions where fire frequency or severity is projected to increase with climate change, fire refugia (areas in the landscape that remain unburned or less affected by fire) will become essential to the persistence of fire‐sensitive species (Meddens et al. 2018).

### The role of corridors and connectivity

Because even the largest core areas are of insufficient size to sustain populations of wide‐ranging carnivore species, ensuring habitat connectivity between protected areas is a major element of rewilding (Soulé & Noss 1998) (Table 1). Connectivity conservation has also been identified as a key strategy for enhancing species dispersal and hence persistence under climate change (Heller & Zavaleta 2009). Many species and populations will need to shift their locations to track suitable climatic conditions (Chen et al. 2011). Pearson and Dawson (2005) note that because dispersal to newly suitable habitat may be difficult or impossible under climate change for certain species (especially the plant taxa they focused on), assisted colonization may become a more relevant strategy than conservation of habitat linkages (Table 1).

The relative importance of these two strategies depends on the dispersal ability of particular taxa of concern. Climate velocity, a measure of the rate of dispersal necessary to track climate (Loarie 2009), varies widely across most regions of North America (Carroll et al. 2017). Many species in locations with low climate velocity will be able to disperse to external refugia (newly suitable areas within their dispersal range [Reside et al. 2018]) if connectivity can be maintained between current and future habitat (Table 1) (Keeley et al. 2018).

### Restoration of ecological processes

Although not directly emphasized in the original 3Cs approach, restoration of ecological and evolutionary processes is an inherent element of rewilding because a wild ecosystem is one that maintains natural regimes of stress, disturbance, and stabilizing positive feedbacks (Table 1). Nevertheless, conservation goals based on process restoration have a more complex meaning under climate change (Lorimer et al. 2015). The original proposal for rewilding did not address the fundamental challenge of restoring wildness – or even determining what wildness means – for ecosystems undergoing rapid change toward likely novel states (Soulé & Noss 1998) (Table 1). Although historical baselines will become less relevant as templates for restoration, webs of positive feedbacks remain important for stabilizing composition and structure of communities (Bowman et al. 2015) (Table 1). Restoration of ecological processes may be a key factor in avoiding transition of ecosystems to degraded states due to the interaction of climate change with anthropogenic stressors, such as grazing, logging, and alteration of fire regimes (Noss 2001; Hanberry et al. 2015).

## Example from Yellowstone‐to‐Yukon Region of Integration of Climate Resilience and Rewilding

Our review of the concepts underpinning rewilding suggests that the conservation value of large, connected protected areas persists under climate change but that this unprecedented threat may alter guidelines for the optimal design and placement of reserves. Because a key challenge of climate change exposure stems from its novel geographic patterns, we addressed the question of what planners might do differently when considering climate resilience by summarizing these patterns and their implications for conservation planning in the Y2Y region.

**Figure 2:**
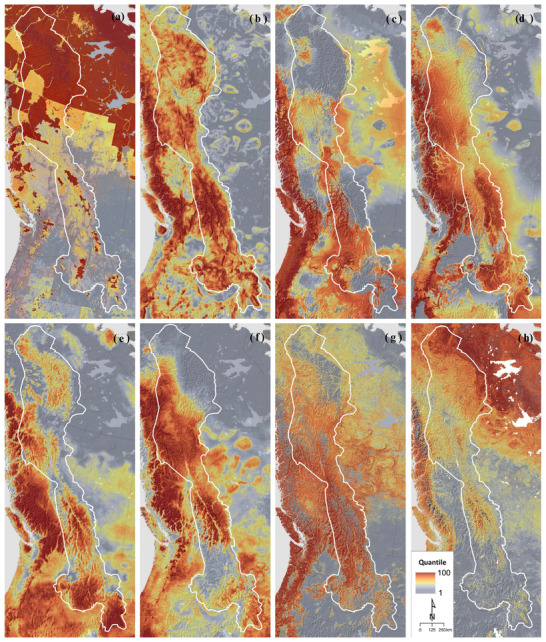
Comparison of 8 metrics relevant to regional climate adaptation planning in the Yellowstone‐to‐Yukon region of western Canada and the Unites States: (a) intactness (inverse of anthropogenic land use intensity), (b) topodiversity (topographic or elevational diversity), (c) refugia based on forward climatic velocity, (d) refugia based on backward climatic velocity, (e) bird species refugia, (f) tree species refugia, (g) aboveground forest carbon, and (h) soil carbon. As in Fig. 1, all data are scaled to equal‐area quantiles for comparability. Sources of data in (a–h) are in Supporting Information.

The Y2Y region, which spans the mountain ranges stretching from Yellowstone National Park in the United States to the Yukon in Canada, is an iconic example of a broad‐scale conservation proposal impelled by the need to conserve a community of wide‐ranging carnivore species (Fig. 1a) (Locke 1994; Carroll et al. 2001). The Y2Y region represents the southernmost extension of intact native assemblages of large carnivores such as the grizzly bear (*Ursus arctos*), wolf, and wolverine (Laliberte & Ripple 2004) for two reasons. First, it encompasses much of the Rocky Mountains, North America's largest north‐south cordillera, which facilitates connectivity with larger boreal carnivore populations. Second, key core areas in the region were protected, primarily for their scenic beauty, before much of the surrounding landscape was modified by Euroamerican settlement (Fig. 1a & 2a) (Locke 1994).

Even a protected area network as extensive as that within Y2Y will experience novel threats to its biota from climate change. Shifts in species distributions due to climate change are already evident in the Y2Y region (Dawe & Boutin 2016), as are ecosystem responses such as landcover change (Wang et al. 2020), including permafrost thaw‐induced boreal forest loss (Carpino et al. 2018). The magnitude of the threat to species and ecosystems from climate change is a function of both climate exposure (how much change in climate a species is likely to experience at a site) and the species’ sensitivity and capacity to adapt to changing climate via evolution, behavioral changes, phenotypic plasticity, or dispersal to new areas (McCarthy et al. 2001). Climate exposure and adaptive capacity can be measured for ecosystems and landscapes as well as for species. Landscape‐scale conservation, by protecting key areas such as climate refugia (Keppel et al. 2015), can increase the adaptive capacity or resilience of a landscape and its ability to retain native species and ecosystems.

### Latitudinal gradients

The key latitudinal gradients influencing climate adaptation planning in the Y2Y region involve climate dissimilarity, microrefugia potential, species diversity, soil carbon, and the human footprint. The most fundamental measure of climate exposure is climate dissimilarity (i.e., how different will the future climate at a location be from its current climate?). A strong latitudinal gradient in dissimilarity is evident, with more rapid warming in boreal regions than at mid‐latitudes (Wang et al. 2020) (Table 2).

**Table 2:** Spatial gradients of climate exposure and related metrics in the Yellowstone‐to‐Yukon region and their implications for conservation planning

Type of gradient		Direction of increase		
Latitudinal	Metric	south to north	Conservation planning implication	Generality^*^
	climate dissimilarity (temperature)	→	Larger reserves will be required to ensureclimate resilience in boreal region than inmid‐latitude regions.	+
	elevation and topodiversity	←	Boreal climate refugia may be driven by ecological features rather than topography.	‐
	species richness	←	Conservation strategies based on representation (i.e., hotspots) are more relevant in mid‐latitudes than in boreal region.	+
	human footprint	←	This gradient underlies the ability of the Yellowstone‐to‐Yukon region and other longitudinal cordilleras to support carnivore populations.	+
	soil carbon	→	Peatlands in interior boreal provide both refugia and carbon storage benefits.	∼
Longitudinal		maritime to continental		
	climate connectivity role	→	Protection of eastern slope of cordillera enhances climate connectivity.	∼
	species richness	←	A hotspot strategy is more relevant in the maritime versus continental sections of the region.	∼
	aboveground carbon	←	Mid‐montane mesic forests can provide important benefits as stores of aboveground carbon.	+
Elevational		low to high		
	topodiversity	→	Montane protected areas have an important role in providing microrefugia.	∼
	forward climatic velocity	→	Alpine areas will experience high species turnover.	+
	backward climatic velocity	←	Mid to upper montane areas provide important macrorefugia. Interior basin ecosystems will experience high climate exposure, with species loss and ecosystem process disruption.	+

^*^Level of generality: +, high; ‐, low; ∼, intermediate.

Within Y2Y, both elevation and topographic diversity generally increase from north to south (Fig. 2b & Table 2) (See Supporting Information for spatial data references and Beckers and Carroll [2020] to view and download data.) The relatively low local topographic diversity (and hence low potential for topographic microrefugia) in much of boreal Y2Y compounds the effect of high climate dissimilarity and exposure there. A gradient of decreasing species diversity with latitude is also evident within the Y2Y region (Table 2). Metrics based on climatic‐niche models for individual species therefore project that midlatitude areas will provide refugia for a greater number of species than will boreal regions (Stralberg et al. 2018).

The several latitudinal gradients collectively imply several guidelines for climate adaptation planning. Topographically complex boreal landscapes such as the Mackenzie Mountains assume importance due to their rarity, as do ecologically driven refugia, such as peatland complexes and lake margins (Stralberg et al. 2020*a*). Recent conservation proposals suggest that protected area networks be expanded to include areas that hold large reserves of aboveground (biomass) and belowground (soil and biomass) carbon, with the aim of reducing disturbances that accelerate release of stored carbon (Dinerstein et al. 2019). Soil carbon is highest in large boreal peatlands, implying that protection of large boreal landscapes in Y2Y and elsewhere is critical for capturing areas of high soil carbon as well as conserving area‐dependent species such as caribou (*Rangifer tarandus*) (Fig. 2b & Table 2) (Hengl et al. 2017; Stralberg et al. 2020*a*).

Anthropogenic pressure, as represented here by the human modification index (Kennedy et al. 2019), forms another fundamental latitudinal gradient in North America, decreasing in intensity from mid‐latitude portions of Y2Y to the boreal region (Fig. 2a & Table 2) (Carroll 2006). The diverse patterns of threat from anthropogenic stressors, such as oil and gas development and forestry, are incompletely represented in the global data set used here and would require further analysis in regional‐scale planning processes.

The rapid increase in anthropogenic development, especially in boreal Y2Y, prompts the question as to whether land‐use change is so immediate a threat that priorities based on climate adaptation goals (e.g., restoration of potential climate refugia in already transformed landscapes) divert resources that would be better spent to protect undeveloped areas irrespective of their climate adaptation value. Although the relative pace of climate versus land‐use change deserves consideration by planners, climate change is projected to alter conservation values even within otherwise undeveloped landscapes and will typically interact with land‐use change to affect biodiversity (Brook et al. 2008). Evidence also suggests that incorporating climate change adaptation and mitigation goals into conservation planning strengthens societal support for protected area expansion (Wright et al. 2019).

### Longitudinal (maritime to continental) gradients

The key longitudinal gradients influencing planning in the Y2Y region involve connectivity under climate change, species diversity, and aboveground carbon. The north–south trending axis of the Rocky Mountains and most other North American ranges allows montane protected area networks, such as Y2Y, to incidentally support latitudinal climate‐driven range shifts. Because organisms will need to avoid hostile climates, dispersal routes between current climate types and where those climates will occur in the future will often be circuitous (Dobrowski & Parks 2016). Thus the distribution of climate corridors (i.e., areas that support climate‐driven dispersal) is driven by complex factors at a range of spatial scales and is not limited to north–south or elevational gradients (Supporting Information) (Carroll et al. 2018). In the Y2Y region, climate corridors occur along the eastern slopes of the central Canadian Rocky Mountains (areas that also served as ice‐free dispersal corridors during the Pleistocene [McDevitt et al. 2009]) and in the valleys of the Mackenzie Mountains and British Columbia's Inland Temperate Rainforest (Table 2 & Supporting Information).

A strong longitudinal precipitation gradient is produced by predominantly west‐to‐east atmospheric circulation interacting with the north–south Rocky Mountain cordillera. The distribution of the taxonomic groups we examined, particularly tree species, shows a longitudinal gradient driven by precipitation patterns, with western, mesic areas supporting higher species diversity (Stralberg et al. 2018) (Fig. 2g & Table 2). These mesic forested areas also support the region's highest levels of aboveground carbon (Table 2) (Santoro 2018).

In other regions, such as the Pacific coast of Canada, areas with high levels of below‐ and aboveground carbon may overlap to a greater degree than is seen within Y2Y (Buotte et al. 2020).

### Elevational gradients

Montane ecosystems of Y2Y show elevational gradients in climate exposure, with rapid change predicted in alpine areas when a full suite of temperature and precipitation metrics are considered (Carroll et al. 2017). The high elevational and topographic diversity of the southern and central Y2Y region, however, should allow microrefugia to play a role in buffering such changes (Keppel et al. 2015) (Table 2). Although some populations will be able to persist in microrefugia because of their capacity to tolerate expected climatic shifts via genetic or behavioral adaptation, many populations will need to shift broadly in space.

Climate velocity, the speed at which an organism needs to travel to keep pace with climate, can be measured by categorizing climate into types and measuring the straight‐line distance between a site and the nearest site with the same climate type in a different period (Hamann et al. 2015). Forward climatic velocity, based on the distance between a site's current climate type and the nearest site with the same climate type under future climates, represents the rate at which an organism currently at a location must move to find future suitable climate (Hamann et al. 2015). Backward climate velocity, based on the distance between a site's future climate type and the nearest site with the same climate type under current climates, represents the rate at which organisms adapted to a location's future climate will need to move to colonize that location (Hamann et al. 2015).

Forward velocity, which provides information on the ability of resident species and ecosystems to persist regionally, will often be high in alpine areas because reaching the nearest analogous future climate may require dispersal to distant mountaintops (Fig. 2c & Table 2). The high forward velocity of Y2Y's upper montane areas, especially high latitude ranges such as the Mackenzie Mountains, suggests these areas will experience substantial species turnover and ecosystem shifts. Backward velocity, which reflects a location's ability to serve as a refugium for species, is often high in valley bottoms because organisms must travel longer distances to colonize these locally new habitats (Fig. 2d & Table 3). Conversely, the low backward velocity of Y2Y's alpine and upper montane areas suggests their role as refugia for species from adjacent downslope areas. Nevertheless, an effective climate resilience strategy built around high‐elevation protected areas must also encompass complete elevational gradients, including low‐elevation areas currently underrepresented in the protected area network (Scott et al. 2001).

Patterns of species range shifts under climate change are complex (Rapacciuolo et al. 2014; Lenoir & Svenning 2015). Because climate velocity is influenced by processes operating at multiple scales (e.g., a site's local and regional topographic position and its location in relation to global climate circulation patterns), patterns shown by velocity metrics are more informative than simpler rules of thumb, such as movement upslope or to higher latitudes. Multivariate velocity metrics, such as we considered here, also reflect the understanding that future climate projections suggest low alignment between shifts in temperature and precipitation, with consequent challenges for species attempting to track climatic niches (Oldfather et al. 2020).

### Biogeographic patterns and gradients

Climate velocity and similar metrics based on nonspecies‐specific data represent a coarse‐filter surrogate used to inform conservation of the vast majority of taxa for which detailed information is lacking (Noss & Cooperrider 1994). Ideally, however, such data should be complemented with fine‐filter metrics focused on individual species, where that information is available (Tingley et al. 2014). Biotic velocity is a fine‐filter metric based on correlations between species distributions and current climatic conditions, which are then projected forward to predict distribution under future climates (Carroll et al. 2015). Biotic velocity represents the distance between a site and the nearest site projected to be climatically suitable for the species under projected future climates.

In comparison with refugia defined solely on the basis of climatic data, the distribution of biotic refugia in Y2Y is additionally influenced by biogeographic factors that have made certain regions more biodiverse than expected based on climate alone. Macrorefugia for tree species are primarily found in the Greater Yellowstone Ecosystem, the Central Canadian Rockies, and the western edge of the Y2Y region in British Columbia, which receives relatively more maritime influence (Fig. 2g) (Stralberg et al. 2018). Important songbird refugia occur in montane southern Y2Y and northern areas such as the Mackenzie Mountains (Fig. 2f). Future research is needed to expand available data on biotic velocity in Y2Y beyond the two major taxonomic groups (trees and songbirds) we reviewed (Stralberg et al. 2018).

In temperate and boreal latitudes, the biased placement of existing protected areas in low‐productivity areas often reduces their overlap with hotspots of species diversity and endemism (Scott et al. 2001). Biotic‐velocity‐based refugia metrics, because they assign additional weight to biodiverse areas, prioritize areas with a broad elevational range (Stralberg et al. 2018). Coarse‐filter goals based on protection of climatic‐velocity‐based refugia and corridors, by aiming to represent all climate types, also distribute conservation priorities beyond the montane and arid regions that hold many existing protected areas.

Starplots provide a useful tool for comparing the relative intensities of climate exposure in different subregions or protected areas (Garcia et al. 2014; Beckers & Carroll 2020). Comparison of starplot patterns for 7 of Y2Y's major protected area complexes illustrates the combined effect of the gradients described above (Fig. 1b). The contrast in starplot patterns between southern and northern protected areas within Y2Y (Fig. 1b) reflects the long‐noted dichotomy between centers of species diversity (hotspots) and wild landscapes (coldspots) (Kareiva & Marvier 2003). Northern protected areas within Y2Y score highly in intactness and protection of soil carbon, whereas southerly protected areas play a greater role in providing macrorefugia as defined by both species models and climate velocity (Fig. 1b).

### Generality of gradients

The specific spatial gradients we identify within Y2Y vary in their generality to other regions globally (Table 2). For example, a large proportion of the arid southwestern United States lies within protected areas that hold centers of species diversity and endemism that are expected to transition to novel climates in the coming decades (Carroll et al. 2017). Many of these, such as Grand Canyon National Park, encompass substantial elevational gradients but not the broad latitudinal gradients in the Y2Y protected area network, which may heighten the vulnerability of their biota to climate change. The generality of lessons from Y2Y may also be reduced in regions of low topographic relief, such as the boreal and mid‐latitude plains of North America, where broad‐scale connectivity initiatives to enhance climate resilience may focus on ecologically driven refugia such as peatland complexes rather than topographic features (Stralberg et al. 2020*a*).

Patterns of projected climate change suggest that many megadiverse regions in North America that served as paleorefugia under past cooling, such as upper montane areas at the southern end of major north–south ranges (e.g., southern Appalachians and Sierra Madre Occidental), are projected to experience extremely high climate exposure (i.e., forward velocity) under future warming (Carroll et al. 2017). An expanded view of rewilding that gives explicit recognition to maintenance of intact ecosystem processes and stabilizing feedbacks, such as fire‐vegetation loops, will be critical in helping increase the resilience of these regions to climate change.

Although Y2Y's patterns of climate exposure are not universal, a key aspect of climate change is that relatively consistent spatial patterns characterize the geography of climate exposure. A qualitative understanding of these patterns, in addition to the high‐level planning concepts reviewed earlier and place‐specific priorities derived from detailed mapping (Stralberg et al. 2020*b*), can help planners craft conservation strategies that are more resilient to uncertainty regarding the future intensity of climate change.

### Planning under uncertainty

Because the rate at which humanity will reduce future emissions of greenhouse gasses is unknown, the pace and magnitude of climate change is inherently uncertain. Therefore, resilient strategies are needed for conserving conservation targets under a range of potential future climate change trajectories. At broad regional and continental extents, conservation prioritizations based on the location of climate connectivity areas have proved relatively robust to alternate future climate scenarios (Carroll et al. 2018). However, regional and local conservation planning processes benefit from incorporating quantitative methods that explicitly account for uncertainty (Moilanen et al. 2006), as well as qualitative guidelines for factoring uncertainty into site‐level management strategies (Belote et al. 2017). More generally, the establishment and restoration of protected areas and connectivity to facilitate adaptive dispersal and protect climatic refugia is in large part a no‐regrets strategy that serves other societal goals beyond ameliorating the effects of climate change.

## Conclusion

Conservation scientists have called for expansion of the global protected area network and more effective placement of new protected areas as critical measures to counter threats arising from the twin crises of accelerating climate change and species extinctions (Dinerstein et al. 2019). A key step in increasing the effectiveness of such expansion is a better understanding of how threats to biodiversity from climate change alter the conceptual underpinnings of rewilding and other ambitious goals for large interconnected natural areas. We found that previous critiques of the relevance of protected area design principles under climate change (Pearson & Dawson 2005; Araújo 2009) were partially supported (e.g., in recommending an increased focus on environmental gradients), but that key design principles, such as the importance of large core reserves, remain valid.

The Y2Y conservation initiative provides an example of how practitioners can use conceptual rules based on broad‐scale spatial patterns and drivers of threat to inform regional conservation priorities under climate change. Climate‐exposure metrics, such as we describe here, can be key information sources for prioritizing of protection areas that have been identified as macrorefugia, especially in boreal landscapes where climate exposure is greatest (Fig. 1). Many of these areas are currently the focus of conservation planning processes involving the Canadian government and indigenous First Nations.

In more developed landscapes with fewer remaining options for new protected areas, climate resilience can nonetheless be enhanced by expanding existing protection to better represent elevational, latitudinal, and ecosystem gradients. The data on climate exposure we reviewed suggest several rules of thumb regarding the vulnerability of existing protected areas based on their landscape position. Alpine and upper montane areas may experience high levels of climate exposure and loss of native species; montane areas at the southern end of major ranges will be especially affected. Nevertheless, these areas in turn will provide refugia for foothill and lowland species shifting upward in elevation. The eastern slopes of the Rocky Mountains provide an example of a key area for north–south climate connectivity that is poorly represented within existing parks. Additionally, interior basins of western North America are projected to experience high climate exposure and velocity and merit greater conservation attention.

The complexity and uncertainty inherent in projecting future climates has created barriers to consideration of climate change in conservation planning. However, an increasing volume of freely accessible information, applied within “communities of practice” (Wenger 1999) that bring together researchers and practitioners (e.g., staff from governmental agencies, First Nations, and nongovernmental organizations), promises to lower barriers to integrating climate resilience within regional planning processes. Our findings suggest that the unique patterns of threat associated with climate change merit consideration as an additional component of the rewilding within a 4Cs framework for conservation of cores, corridors, carnivores, and climate resilience. Although previous reviews propose that planning for climate adaptation should favor small dispersed reserves, we conclude that climate change strengthens the rationale for networks of large protected areas that represent all landscape types and species, protect intact environmental gradients, and maintain ecological and evolutionary processes, including natural disturbance regimes and stabilizing feedbacks (Noss & Cooperrider 1994).

## Supporting information

Table S1. Sources of data shown in Figs. 1 and 2. Full references provided in main text.
Figure S1. Climate connectivity areas in the Yellowstone‐to‐Yukon region. Red paths indicate areas important (path count >700) for forward climatic connectivity, whereas blue paths indicate areas important for backward climatic connectivity.
