# Supplementary material for: Rewilding in the face of climate change
Source: Conserv Biol. 2020 Jun 1;35(1):155–67. doi: 10.1111/cobi.13531 (PMC7984084; doi:10.1111/cobi.13531)

Supplementary Material for ‘Rewilding in the face of climate change’.

Carlos Carroll[1], Reed F. Noss[2].

1. Klamath Center for Conservation Research, Orleans, CA 95556 USA.

2. Florida Institute for Conservation Science, Melrose, FL 32666 USA.

SI Table S1. Sources of data shown in Figures 1 and 2. Full references provided in main text.

Metric	Source
Intactness (inverse of landuse intensity)	Kennedy et al. 2019
Topodiversity	Carroll et al. 2017
Forward climatic velocity	Carroll et al. 2017
Backward climatic velocity	Carroll et al. 2017
Bird species refugia	Stralberg et al. 2018
Tree species refugia	Stralberg et al. 2018
Aboveground forest carbon	Santoro et al. 2018
Soil carbon (100 cm depth)	Hengl et al. 2017

SI Figure S1. Climate connectivity areas in the Yellowstone-to-Yukon region. Red paths indicate areas important (path count > 700) for forward climatic connectivity, whereas blue paths indicate areas important for backward climatic connectivity. Data from Carroll et al. 2018. Global Change Biology 24:5318-5331.

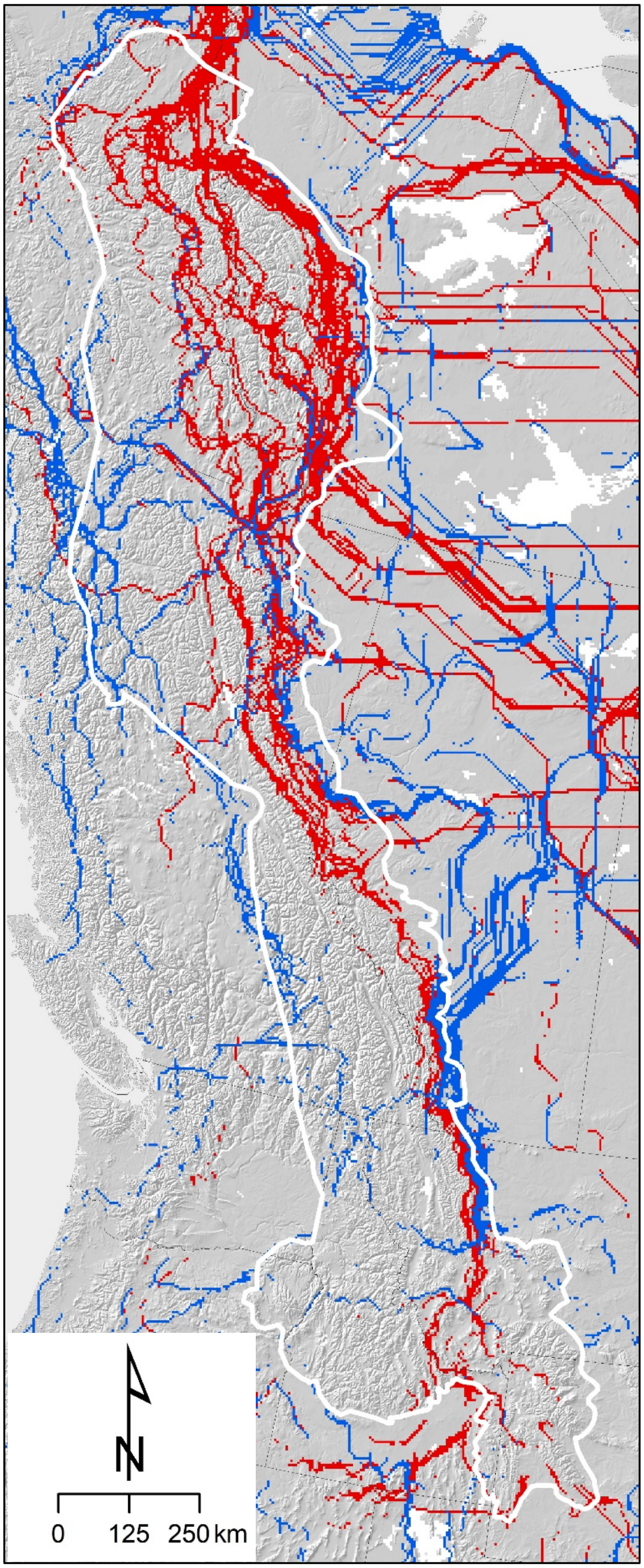

Supplement: Supplementary file 1 — Table S1. Sources of data shown in Figs. 1 and 2. Full references provided in main text. Figure S1. Climate connectivity areas in the Yellowstone‐to‐Yukon region. Red paths indicate areas important (path count >700) for forward climatic connectivity, whereas blue paths indicate areas important for backward climatic connectivity. [file COBI-35-155-s001.pdf]
